# Efficient microbial production of stylopine using a *Pichia pastoris* expression system

**DOI:** 10.1038/srep22201

**Published:** 2016-02-29

**Authors:** Kentaro Hori, Shunsuke Okano, Fumihiko Sato

**Affiliations:** 1 Division of Integrated Life Science, Graduate School of Biostudies, Kyoto University, Kitashirakawa, Sakyo, Kyoto 606-8502, Japan

## Abstract

Stylopine is a protoberberine-type alkaloid that has potential biological activities. Based on the successful microbial production of (*S*)-reticuline, we attempted to produce stylopine from (*S*)-reticuline by the reaction of berberine bridge enzyme, cheilanthifoline synthase (CYP719A5), and stylopine synthase (CYP719A2). Biosynthetic enzyme expression was examined in a methanol-utilizing yeast (*Pichia pastoris*), and both a “consolidated” system with all genes expressed in one cell and a “co-culture” system with three cell lines that each express a single gene were examined. Although both systems efficiently converted reticuline to stylopine, the consolidated system was more rapid and efficient than the co-culture system. However, substrate-feeding experiments revealed a decrease in the conversion efficiency in the consolidated system during successive cultures, whereas the conversion efficiency in the co-culture system remained constant. Thus, the final amount of stylopine produced from reticuline after successive feedings in the co-culture system was more than 150 nmoles from 750 nmoles of (*R, S*)-reticuline (375 nmoles of (*S*)-reticuline). The advantages and drawbacks of the “consolidated” system and the “co-culture” system are discussed.

Stylopine (tetrahydrocoptisine) is a protoberberine-type benzylisoquinoline alkaloid that is found in *Argemone mexicana*^1^. This compound is an intermediate in the biosynthetic pathway of benzophenanthridine alkaloids, such as sanguinarine, and is also a reduced form of coptisine, which is found in *Coptis rhizome*. Although there have been limited pharmacological studies of coptisine and stylopine compared with those on berberine, the physiological activities of protoberberine alkaloids as modulators of lipid metabolism^2^,^3^ have led to an increased demand for methods to prepare more diversified alkaloids such as stylopine and coptisine. However, the presence of two methylene-dioxy rings in the protoberberine skeleton of stylopine makes chemical synthesis difficult. Thus, a method that relies more on a biotechnological approach for production is required.

Although stylopine is an intermediate in the benzophenanthridine alkaloid biosynthesis, it shows only scant accumulation in cultured cells compared with the accumulation of coptisine, an oxidized form of stylopine, because stylopine is so easily oxidized^4^,^5^. As major parts of the biosynthetic pathway for sanguinarine from tyrosine have been characterized at the molecular level (Fig. 1), there are several possible options for the production of stylopine using biotechnological techniques. For example, the RNA silencing of the gene for an enzyme that is downstream of stylopine in sanguinarine biosynthesis, i.e., tetrahydroprotoberberine *N*-methyltransferase, is feasible, as shown for the successful accumulation of reticuline in transgenic *Eschscholzia californica* cells with an RNA interference (RNAi) vector for berberine bridge enzyme (BBE) to convert reticuline to scoulerine^6^. However, the construction of transgenic plant cells with stable productivity is both time-consuming and difficult due to the instability of cultured cells.

In this study, we examined a much simpler method for producing stylopine from reticuline in microbial cells, as we had recently established an efficient system for producing reticuline from a simple carbon source such as glucose/glycerol in *Escherichia coli*^7^. Stylopine is synthesized from (*S*)-reticuline in a reaction consisting of three steps, i.e., berberine bridge enzyme (BBE)^8^, cheilanthifoline synthase (e.g., CYP719A5 from *E. californica*, CHS)^9^, and stylopine synthase (e.g., CYP719A2/A3 from *E. californica*, STS)^10^; however, a previous report indicated that the BBE step was inefficient for expression in a microbial system, especially in yeast^11^.

Although reconstruction of the entire pathway in single cells is preferred due to the simplicity of handling and management, we first examined the stepwise optimization of the pathway to overcome the inefficiency of BBE. Next, we examined the reconstruction of the pathway through the co-culturing of each biosynthetic reaction. We also examined the co-expression of the genes for all of the biosynthetic enzymes in a single cell as a consolidated form and compared the efficiency of the biosynthesis with that in the co-culture (Supplementary Fig. S1).

In this study, we examined the efficacy of *Pichia* cells for the high expression of cytochrome P450s, as the expression level of this membrane protein is usually low and not sufficient for conversion. Fortunately, all three enzymes were successfully expressed in *Pichia* cells. Thus, both the co-culture of the transformants with each step and the consolidated transformant showed the efficient (more than 80%) conversion of reticuline to stylopine. The advantages of the consolidated transformant, which showed very rapid conversion within 2 hr, are discussed along with those of the co-culture system, which offers greater flexibility for pathway design and the potential to avoid metabolic interference among the biosynthetic enzymes.

## Results

### Comparison of yeast expression systems

Before we examined the reconstruction of the biosynthetic pathway, we explored the efficacy of Pichia cells as a host, as budding yeast (*Saccharomyces cerevisiae*), although useful in other scenarios, shows low expression of recombinant proteins. In fact, Pichia is known for the high expression of recombinant proteins when using a strong inducible promoter of alcohol oxidase (AOX). In addition, Pichia cells are easy to use for the stable integration of multiple transgenes into the genome.

Although budding yeast and Pichia require different conditions for the expression of recombinant proteins, we simply compared the conversion efficiency based on the cell number. Approximately 3 × 10^7^ cells (about OD_600_ = 15) with the *Ec*CYP719A5 gene were suspended in 2-[4-(2-hydroxyethyl)-1-piperazinyl] ethane sulfonic acid (HEPES) buffer with 200 μM scoulerine and incubated at 30 °C, 200 rpm, for 24 hr. An analysis of the reaction product by liquid chromatography-mass spectrometry (LC-MS) clearly indicated a several-fold higher conversion of scoulerine into product (cheilanthifoline) in Pichia cells (70% conversion) compared to budding yeast (20% conversion) (Supplementary Fig. S2). Thus, further experiments were carried out with Pichia cells. Because our preliminary study also indicated that Pichia cells incubated in buffered methanol-complex medium (BMMY medium) showed enhanced enzyme activity (Supplementary Fig. S3), further biosynthesis experiments were performed in BMMY medium.

### Stylopine production by co-culture of *P. pastoris* with a single gene construct

Transgenic Pichia cells with a single gene construct for BBE, CYP719A5, or CYP719A2 were prepared, and their bioconversion activities were determined by LC-MS analysis of the reaction products from appropriate substrates (Fig. 2). The formation of cheilanthifoline by CYP719A5 from scoulerine was confirmed by the production of stylopine by CYP719A2 from the reaction product of CYP719A5. The by-product of the CYP719A2 reaction (m/z 320) was determined to be coptisine by comparing with an authentic sample (Fig. 2G,I, and Supplementary Fig. 4). The accumulation of coptisine would be due to the spontaneous oxidation of stylopine or endogenous yeast enzymes. These analyses showed that each enzyme had sufficient bioconversion activity, and the reaction products of BBE and CYP719A5 (i.e., scoulerine from reticuline and cheilanthifoline from scoulerine) were secreted into the culture medium, whereas the stylopine and coptisine produced by CYP719A2 were mainly retained in the cells (Fig. 2). Based on this observation, we conducted a sequential bioconversion from reticuline to stylopine using the co-culture of *P. pastoris* cells that expressed each biosynthetic enzyme.

As shown in Fig. 3, reticuline was taken up by Pichia cells within 24 hr and converted into scoulerine, cheilanthifoline, stylopine, and coptisine. During the subsequent 24 hr of incubation, scoulerine became undetectable in both the medium and cells, whereas low levels of cheilanthifoline were still seen in the medium, and the levels of stylopine and coptisine increased. After another 24 hr of incubation (i.e., 72 hr of incubation in total), the cheilanthifoline also became undetectable, whereas there was very little change in the amount of stylopine and a slight increase in coptisine. Finally, approximately 33 nmoles of stylopine and 22 nmoles of coptisine were produced from 150 nmoles of (*R, S*)-reticuline. This result clearly indicates that the co-culture of Pichia transformants with a single enzyme gene for 3 successive reactions effectively reconstituted the reaction *in vitro* to produce stylopine from reticuline. Because the first-step enzyme BBE is known to react specifically with (*S*)-reticuline^8^,^12^,^13^, most of the remaining reticuline should be (*R*)-reticuline. When only (*S*)-reticuline in a racemic substrate ((*R, S*)-reticuline) was used in this biosynthesis of stylopine and coptisine, the conversion efficiency was calculated to be approximately 80%. This high conversion efficiency is superior to that reported previously (up to 11%)^11^.

### Stylopine production using Pichia with a consolidated expression construct

Although the co-culture of Pichia cells with a single biosynthetic enzyme gene showed the highly efficient conversion of reticuline into stylopine and coptisine, the full conversion required 72 hr. Because co-culturing requires the transport of substrates among cells, we sought to further improve the bioconversion efficiency through the co-expression of all three genes in single cells (consolidated system). To express the three genes in a single cell, they were cloned in pAO-815 vector (Supplementary Fig. S1) and then stacked in a single vector, as described in the Materials and Methods. The resulting consolidated pAO-815 vector, which contained the genes for BBE, CYP719A5, and CYP719A2, was transformed into *P. pastoris* strain GS115. Although the transformation efficiency decreased with an increase in vector size (greater than 15 kb), a transformant was obtained. The bioconversion activity of this transformant (B52#1) was further characterized and compared with the results obtained using the co-culture system.

As shown in Fig. 4, 150 nmoles of (*R, S*)-reticuline were converted into stylopine and coptisine after only 2 hr of incubation. The accumulation of intermediates, i.e., scoulerine (approximately 20 nmoles) and cheilanthifoline (approximately 3 nmoles), was only transiently detected at 2 hr of incubation. The final conversion rate of (*S*)-reticuline to stylopine and coptisine was almost 80% when only (*S*)-reticuline was used as a substrate, as mentioned above. This large improvement in the conversion efficiency is likely due to the lack of a need to transport metabolites across cell membranes, which separate the individual reactions in the co-culture system. Again, stylopine was mainly retained in the cells and only approximately 1/8 of the stylopine produced was secreted into the medium. Coptisine was only detected in the cells.

### Optimization of stylopine production

Because the consolidated expression system showed a high conversion efficiency, we attempted to increase the stylopine production by substrate feeding. Thus, we added an equimolar amount of substrate (150 nmoles (*R, S*)-reticuline) to the consolidated reaction mixture five times every 2 hr (0, 2, 4, 6, 8 hr). Substrate feeding increased the production of stylopine and coptisine (their maximum amounts were approximately 85 nmoles and 54 nmoles, respectively, after 8 hr of incubation), and a considerable amount of scoulerine remained in the mixture without being converted (Fig. 5A). As shown in Fig. 5D, the bioconversion rate of scoulerine into cheilanthifoline gradually decreased, unlike the other rates.

Because the BBE reaction generates H_2_O_2_^14^, we examined the effect of an antioxidant, ascorbic acid (final 30 mM). Interestingly, the bioconversion rate of scoulerine was only minimally improved, whereas the BBE activity showed considerable improvement, and more stylopine was produced than in the absence of the ascorbic acid treatment (Fig. 5B,D). The addition of ascorbate also affected stylopine oxidation, and the coptisine was no longer detectable.

These results suggest that the H_2_O_2_ generated by BBE impaired the activities of the other biosynthetic enzymes. The CYP719A5 activity would be the most sensitive and was not protected by the addition of ascorbate. To solve this problem, we performed a substrate-feeding experiment with the co-culture system. Because the co-culture system needs more time to convert the substrate, we added an equimolar amount of substrate (150 nmoles (*R, S*)-reticuline) to the reaction mixture five times every 24 hr (0, 24, 48, 72, 96 hr). The co-culture system with substrate feeding showed a continuous increase in stylopine production over the five successive feedings, and only a small amount of scoulerine was detected (Fig. 5C). This result clearly indicates that the bioconversion activity of CYP719A5 is greatly improved in the co-culture system with substrate feeding (Fig. 5D). The co-culture ultimately finally produced 170 nmoles of stylopine from 750 nmoles of (*R, S*)-reticuline (375 nmoles of (*S*)-reticuline) after 120 hr of incubation and five substrate feedings.

## Discussion

A microbial system to produce stylopine from reticuline with more than 90% efficiency within 2 hr of incubation was established in Pichia yeast. The use of a strong inducible promoter of the AOX gene and consolidated co-expression in a single cell was an efficient strategy for rapid conversion. However, the co-culture of single-gene transformants was also effective, although a longer cultivation period was required than with the consolidated system. Although the consolidated system clearly offers a rapid and efficient conversion of metabolites in the reaction, further studies will be needed on metabolic channelling among the expressed enzymes. Even if there is no metabolic channelling, the lack of a need for the transport of metabolites across cell membranes is both energy cost-effective and time-saving for biosynthesis.

By contrast, the substrate-feeding experiment revealed that in the consolidated system, the stylopine production decreased with time and with an increase in the substrate concentration. This problem was caused by a decrease in the enzyme activity of CYP719A5, which converts scoulerine into cheilanthifoline. The increase in the BBE activity under the addition of ascorbate suggests that this enzyme may be highly sensitive to active oxygen. Interestingly, this decrease in the CYP719A5 activity in the co-culture system was not observed with the application of five successive substrate feedings. This result suggests that in some cases, the separation of biosynthetic enzymes may be needed to maintain high efficiency. Although the co-culture system requires a much longer time for the complete reaction, this longer time is offset by the improved enzyme stability, as shown in our experiment.

**Figure 1:**
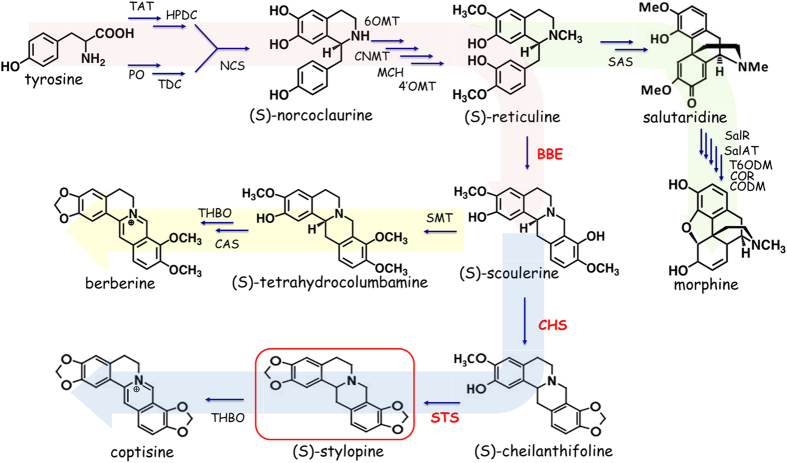
Isoquinoline alkaloid biosynthetic pathway. TAT, tyrosine aminotransferase; HPDC, 4-hydroxyphenylpyruvate decarboxylase; PO, polyphenol oxidase; TDC, tyrosine decarboxylase; NCS, norcoclaurine synthase; 6OMT, norcoclaurine 6-*O*-methyltransferase; CNMT, coclaurine *N*-methyltransferase; MCH, *N*-methylcoclaurine 3′-hydroxylase; 4′OMT, 3′-hydroxy-*N*-methylcoclaurine 4′-*O*-methyltransferase; BBE, berberine bridge enzyme; CHS, cheilanthifoline synthase; STS, stylopine synthase; SMT, scoulerine 9-*O*-methyltransferase; CAS, canadine synthase; THBO, tetrahydroberberine oxidase; SAS, salutaridine synthase; SalR, salutaridine reductase; SalAT, salutaridinol acetyltransferase; T6ODM, thebaine 6-*O*-demethylase; COR, codeinone reductase; CODM, codeine 3-*O*-demethylase.

The co-culture system also provides more flexibility in terms of biocatalysts, i.e., yeast cells expressing biosynthetic enzymes. For example, in the co-culture system, it is much easier to modify the incubation conditions with different cell densities of each transformant or different ratios of the combined transformants, as shown in Supplementary Fig. S5; that is, we could adjust the enzyme activities in the reaction without modifying the promoter strength of the gene constructs, whereas our initial condition was almost optimal for our bioconversion. Furthermore, the combination of each BBE-expressing, scoulerine 9-*O*-methyltransferase-expressing and *Cj*CYP719A1-expressing Pichia cell can produce tetrahydrocolumbamine from reticuline without the time-consuming construction of an expression construct and transformation of Pichia cells (Fig. 1). This flexibility is a major advantage of the co-culture system.

Another breakthrough in our system is that there is no need for supplementation with CPR (cytochrome P450 reductase), whereas some budding yeast systems require the additional expression of CPR^11^. However, no need for supplementation with CPR does not deny the possibility that the expression of plant-derived CPR may improve P450 activity, as P450 was expressed at very low levels in Pichia, as determined by Coomassie Brilliant Blue staining after sodium dodecyl sulfate-polyacrylamide gel electrophoresis. Furthermore, because the introduced gene was stably integrated into the Pichia genome, the collection of transformant cells would be useful as a toolbox for the future design of biosynthetic pathways for the production of novel chemicals. In fact, all of the Pichia transformants that were used in this study maintained their activity for more than 10 months without freezing.

In conclusion, we established a microbial system for producing a protoberberine-type alkaloid in Pichia cells. The consolidated and co-culture systems used here both have advantages and disadvantages and both may be useful for the design of pathways for the expression of plant enzyme genes to produce target compounds.

## Materials and Methods

### Chemicals

(*R, S*)-reticuline and (*S*)-scoulerine were gifts from Mitsui Chemicals, Inc. (Tokyo, Japan). Stylopine was from the collection of Dr. K. Iwasa. Coptisine was purchased from Wako Pure Chemical Industries, Ltd. (Osaka, Japan).

### Construction of the Pichia expression vector

Expression constructs for the three biosynthetic enzyme genes were prepared using nucleotide sequence information for *E. californica* (*BBE*; S65550, *CYP719A5*; AB434654, and *CYP719A2*; EU882969)^8^,^9^,^10^. Although the two biosynthetic enzymes CYP719A2 and CYP719A3 were identified as stylopine synthase in *E. californica*, we used CYP719A2 for stylopine production because of its high substrate specificity^10^. All genes were chemically synthesized by GenScript Japan, Inc. to optimize the codon usage for Pichia cells with the extension of a *Spe* I site for ligation. After digestion with *Spe* I, the DNA fragments were inserted into an *Avr* II site of the pPIC3.5 vector (Invitrogen, Carlsbad, CA, USA). After construction of the vectors, no mutation was confirmed by sequencing.

A consolidated construct to express all three genes (i.e., codon-optimized *BBE*, *CYP719A5*, and *CYP719A2*) was prepared in the pAO815 vector according to the User Manual for the Multi-Copy Pichia Expression Kit (Invitrogen). First, each gene was inserted in the pAO815 vector, and the construction was confirmed by sequencing. Next, an expression cassette was cut out of the pAO815 vector and stacked to produce a single consolidated vector (Supplementary Fig. S1).

### Transformation of Pichia cells

*Pichia pastoris* strain GS115 cells were transformed with linearized plasmids by electroporation according to the User Manual for the Multi-Copy Pichia Expression Kit (Invitrogen). Transformants were confirmed by colony PCR with gene-specific primers for each construct (Supplementary Table S1). Furthermore, for the co-culture system, transformed Pichia cells that retained a high copy number of the pPIC3.5K plasmid with a single gene (i.e., codon-optimized *BBE*, *CYP719A5*, or *CYP719A2*) were selected on yeast extract peptone dextrose (YPD) agar medium containing 1 mg/ml of the antibiotic G418.

### Expression of recombinant proteins in Pichia cells and evaluation of their bioconversion activities

To confirm the activities of the recombinant enzymes in Pichia, each transgenic Pichia cell containing the gene for *Ec*BBE, *Ec*CYP719A5, or *Ec*CYP719A2 was pre-cultured in YPD medium until OD_600_ = 30. Thereafter, each yeast cell was recovered from 1.5 ml of each culture medium and re-suspended individually in 1.5 ml BMMY medium, which was composed of 1% w/v yeast extract, 2% w/v peptone, 1.34% w/v yeast nitrogen base, 100 mM potassium phosphate, pH6.0, 4 × 10^−5^% w/v biotin, and 0.5% v/v methanol. After the induction of recombinant protein expression in BMMY medium for 24 hr, 0.5% v/v methanol and appropriate substrate were added (i.e., reticuline for BBE, BBE reaction product (scoulerine) for CYP719A5, and CYP719A5 reaction product (chielanthifoline) for CYP719A2 reaction) for an *in vivo* reaction at 30 °C. After 24 hr, the reaction products were analysed by an LC-MS system.

### Re-construction of the stylopine biosynthetic pathway using Pichia cells

In the co-culture system, high-copy Pichia transformants containing the *Ec*BBE, *Ec*CYP719A5, or *Ec*CYP719A2 gene were pre-cultured separately in YPD medium until OD_600_ = 30. Thereafter, each Pichia cell was harvested from 0.5 ml culture medium and then re-suspended in 1.5 ml BMMY medium. After induction for 24 hr with 0.5% v/v methanol, 150 nmoles (*R, S*)-reticuline (final concentration 100 μM) were added to start the *in vivo* reaction at 30 °C. Every 24 hr, 0.5% v/v methanol was added, and the reaction products were analysed by an LC-MS system.

In the consolidated system, transgenic Pichia cells containing all three genes were pre-cultured in YPD medium until OD_600_ = 30. An *in vivo* reaction was then started after 24 hr of incubation with 0.5% v/v methanol in 1.5 ml BMMY medium by the addition of (*R, S*)-reticuline. Reaction products were analysed at 2, 4, 8, and 16 hr of incubation after the addition of (*R, S*)-reticuline by an LC-MS system.

### LC-MS analysis of alkaloids

**Figure 2:**
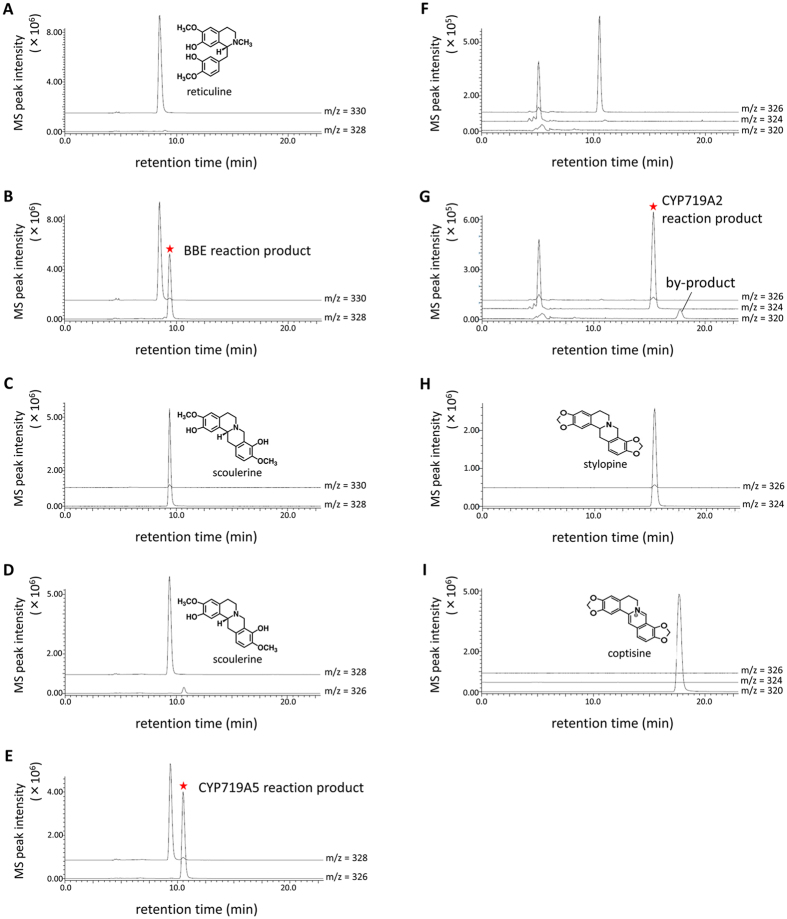
Bioconversion activity of Pichia cells with single BBE, CYP719A5, or CYP719A2 expression construct. (**A**) Vector control reaction with reticuline (m/z330, [M + H]^+^), (**B**) BBE reaction with reticuline, (**C**) authentic scoulerine (m/z 328, [M + H]^+^), (**D**) vector control reaction with scoulerine (m/z 328, [M + H]^+^), (**E**) CYP719A5 reaction with scoulerine (m/z 328, [M + H]^+^), (**F**) vector control reaction with CYP719A5 reaction product, (**G**) CYP719A2 reaction with CYP719A5 reaction product, (**H**) authentic stylopine (m/z 324, [M + H]^+^), and (I) authentic coptisine (m/z 320, [M]^+^). (**A,B,D**,**E**) are LC-MS results of supernatant of each reaction mixture, whereas (**F**) and (**G**) are of MeOH extract from incubated Pichia cells.

**Figure 3:**
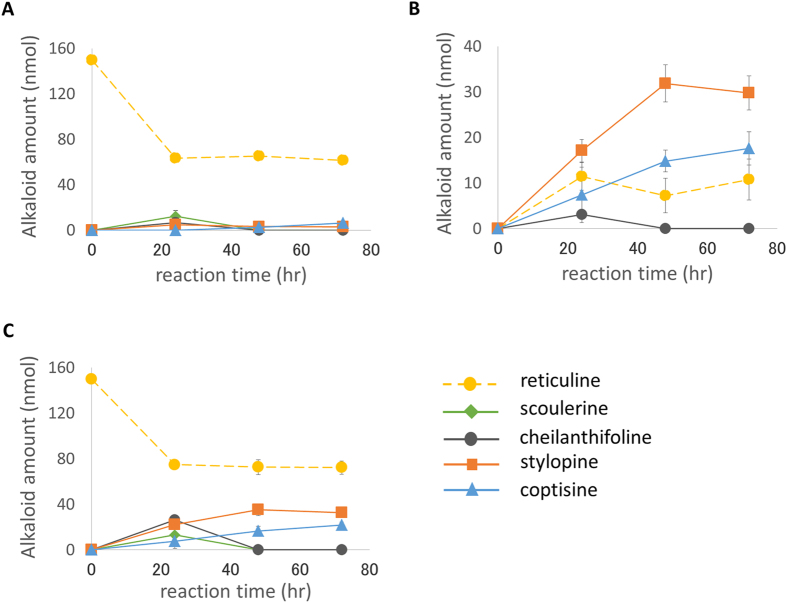
Microbial production of (*S*)-stylopine by co-culture of transgenic *P. pastoris.* Each graph shows the time-dependent transition of the alkaloids included in supernatant (**A**), methanol extract of yeast cells (**B**), and whole reaction mixture (**C**). (*R, S*)-reticuline is a reaction substrate, and the other compounds are products. The amount of each alkaloid was determined from the 280 nm Abs peak height in the LC-MS analysis. Error bars indicate the standard deviation calculated from three independent experiments.

**Figure 4:**
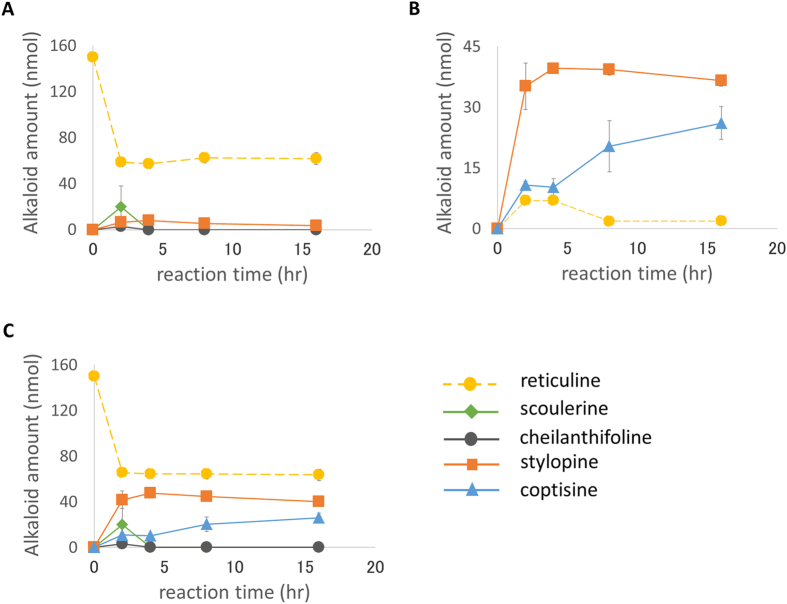
Microbial production of (*S*)-stylopine by the co-expression of biosynthetic enzymes in *P. pastoris*. Each graph shows the time-dependent transition of the alkaloids included in supernatant (**A**), yeast cells (**B**), and whole reaction mixture (**C**). (*R, S*)-reticuline is a reaction substrate, and the other compounds are products. The amount of each alkaloid was determined from the 280 nm Abs peak height in the LC-MS analysis. Error bars indicate the standard deviation calculated from three independent experiments.

**Figure 5:**
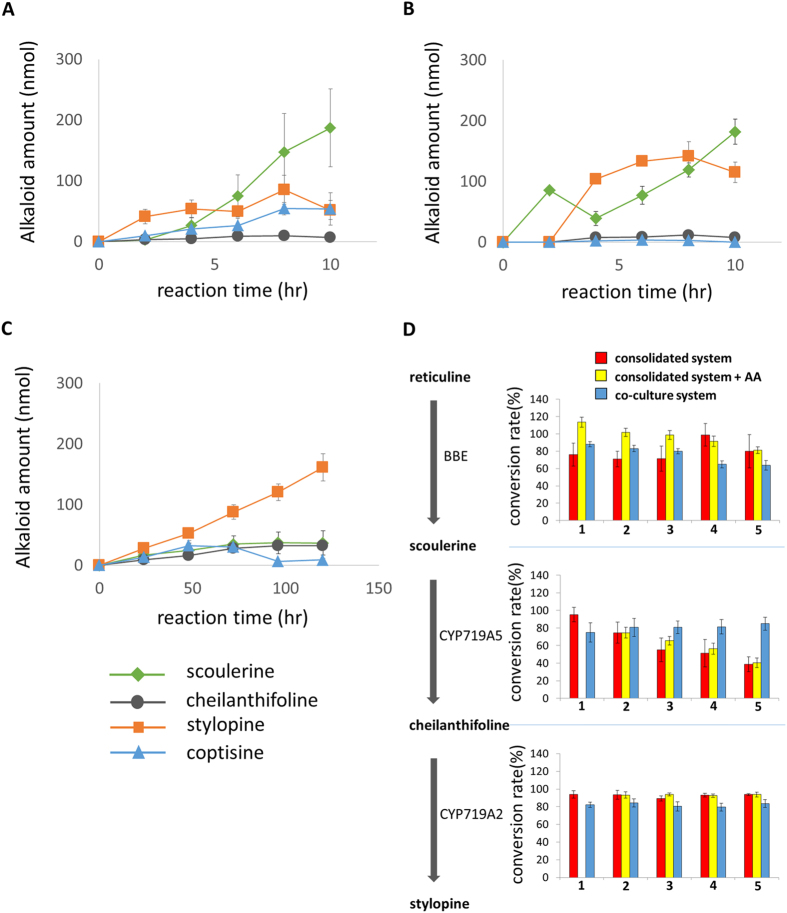
Comparison of bioconversion activities between the consolidated and co-culture production systems with substrate feeding. Each graph shows the changes in the alkaloids in the whole reaction mixture; (**A**) consolidated system; (**B**) consolidated system + ascorbic acid (AA); (**C**) co-culture system. The bioconversion rates for each step of the reaction were calculated based on the change in the amount of each alkaloid (**D**). The amount of each alkaloid was determined from the 280 nm peak height in the LC-MS analysis. Error bars indicate the standard deviation calculated from three independent experiments.

Alkaloids produced by the *in vivo* reaction were analysed by an LC-MS system. *Ec*BBE, *Ec*CYP719A5, and *Ec*CYP719A2 activities in Pichia cells (Fig. 2) and stylopine production from (*R, S*)-reticuline (Figs 3, 4 and 5) were analysed with an LC-MS 2020 system (SHIMADZU, Kyoto, Japan; ESI-MS at 1.5 kV, positive ion mode); UV detection, absorbance measurement at 280 nm with an SPD-20A detector (SHIMADZU); column, TSKgel 80-TM (4.6 × 250 mm; TOSOH, Tokyo, Japan); temperature, 40 °C; solvent system, CH_3_CN/H_2_O (4:6, v/v) containing 1% AcOH and flow rate 0.5 ml/min. MS fragment spectra of the *in vivo* reaction products of BBE, CYP719A5, and CYP719A2 (Supplementary Fig. S4) were detected by an LC-MS 8030 system (SHIMADZU); ESI-MS, product ion scan mode, m/z 10.00–400.00, collision energy at -35.0 V; UV detection, absorbance measurement at 280 nm with an SPD-M20A detector (SHIMADZU); column, TSKgel 80-TM (4.6 × 250 mm; TOSOH); temperature, 40 °C; solvent system, CH_3_CN/H_2_O (4:6, v/v) containing 1% AcOH and flow rate 0.5 ml/min.

The amount of alkaloids was calculated using the absorbance at 280 nm for each authentic sample, i.e., reticuline, scoulerine, stylopine, and coptisine. The amount of cheilanthifoline was estimated using the product generated from scoulerine based on the assumption that all product was converted into stylopine by *Ec*CYP719A2.

## Additional Information

**How to cite this article**: Hori, K. *et al.* Efficient microbial production of stylopine using a *Pichia pastoris* expression system. *Sci. Rep.* **6**, 22201; doi: 10.1038/srep22201 (2016).

## Supplementary Material

Supplementary Information
