# Supplementary material for: Efficient microbial production of stylopine using a Pichia pastoris expression system
Source: Sci Rep. 2016 Feb 29;6:22201. doi: 10.1038/srep22201 (PMC4770593; doi:10.1038/srep22201)
Supplement: Supplementary Information [file srep22201-s1.pdf]

Supplementary information

Efficient microbial production of stylopine using a *Pichia pastoris* expression system

Kentaro Hori, Shunsuke Okano, Fumihiko Sato*, [1]

[1]Division of Integrated Life Science, Graduate School of Biostudies, Kyoto University, Kitashirakawa, Sakyo, Kyoto 606-8502, Japan

*fsato@lif.kyoto-u.ac.jp

Supplementary Table S1. Sequences of primers used in colony PCR

Straight and dotted lines indicate *Spe* I and *Eco*R I restriction sites, respectively.

primer name	Oligonucleotide sequence (5'- to 3'-)
Primers for pPIC3.5K construct	
Biosynthetic gene-specific primers	
codon-optimized *Ec*BBE pPIC3.5K forward	ACTAGTATGGAAAACAAAACTCCCATCTTC
codon-optimized *Ec*CYP719A5 pPIC3.5K forward	ACTAGTATGGAGGAGTCATTGTGGG
codon-optimized *Ec*CYP719A2 pPIC3.5K forward	ACTAGTATGGAGGAAATGAAAATCTTG
pPIC3.5K vector-specific primer	
3'AOX reverse	GCAAATGGCATTCTGACATCC
Primers for pAO815 construct	
Biosynthetic gene-specific primers	
codon-optimized *Ec*BBE pAO815 forward	GAATTCATGGAAAACAAAACTCCCATC
codon-optimized *Ec*BBE pAO815 reverse	GAATTCCTATATTACAACTTCTCCACCATC
codon-optimized *Ec*CYP719A5 pAO815 forward	GAATTCATGGAGGAGTCATTGTGGGT
codon-optimized *Ec*CYP719A5 pAO815 reverse	GAATTCTTACAATTGGGTTCTAGGAGTGAT
codon-optimized *Ec*CYP719A2 pAO815 forward	ATCGAATTCATGGAGGAAATGAAAATCTTG
codon-optimized *Ec*CYP719A2 pAO815 reverse	GAATTCTTAGTTTCTTCTATTAATTCTAGC

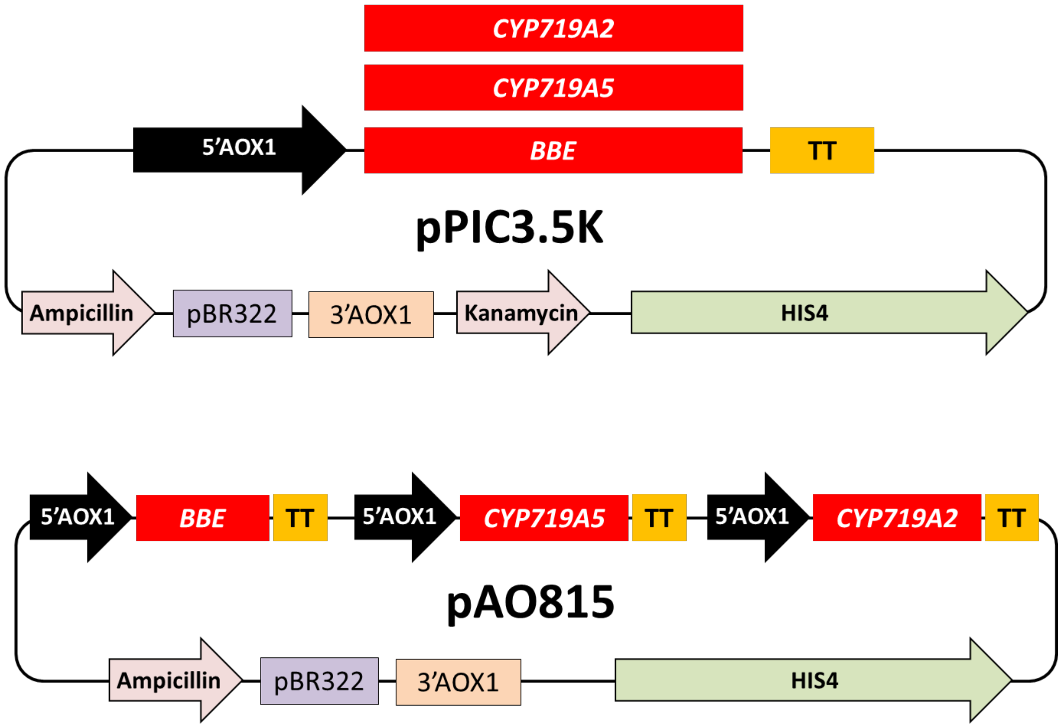

Supplementary Figure S1. Map of vectors for single gene expression (pPIC3.5K) and the co-expression of multiple genes (pAO815).
5'AOX1 = the *AOX1* promoter
TT = Native transcription termination and polyadenylation signal from *AOX1* gene
Ampicillin = Ampicillin resistance gene
pBR322 = *E. coli* origin of replication
3'AOX1 = Sequences from the *AOX1* gene
Kanamycin = Kanamycin resistance gene
HIS4 = *Pichia* wild-type gene coding for histidinol dehydrogenase

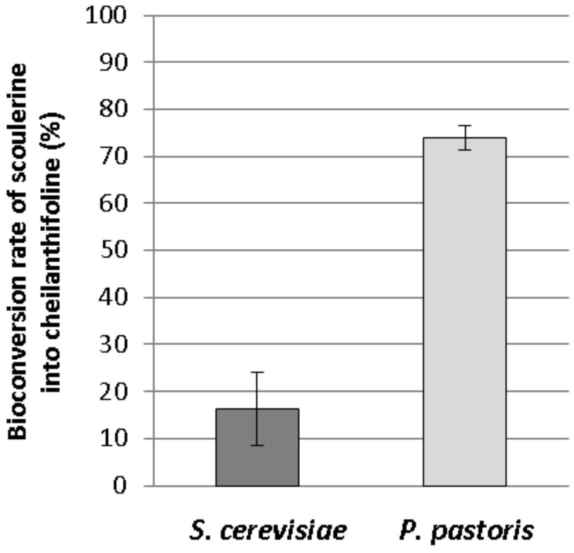

Supplementary Figure S2. Comparison of bioconversion activity in *Saccharomyces cerevisiae* and *Pichia pastoris.*

The rate of the bioconversion of 200 μM scoulerine into cheilanthifoline by *S. cerevisiae* and *P. pastoris* cells expressing CYP719A5. Error bars indicate the standard deviation calculated from three independent experiments.

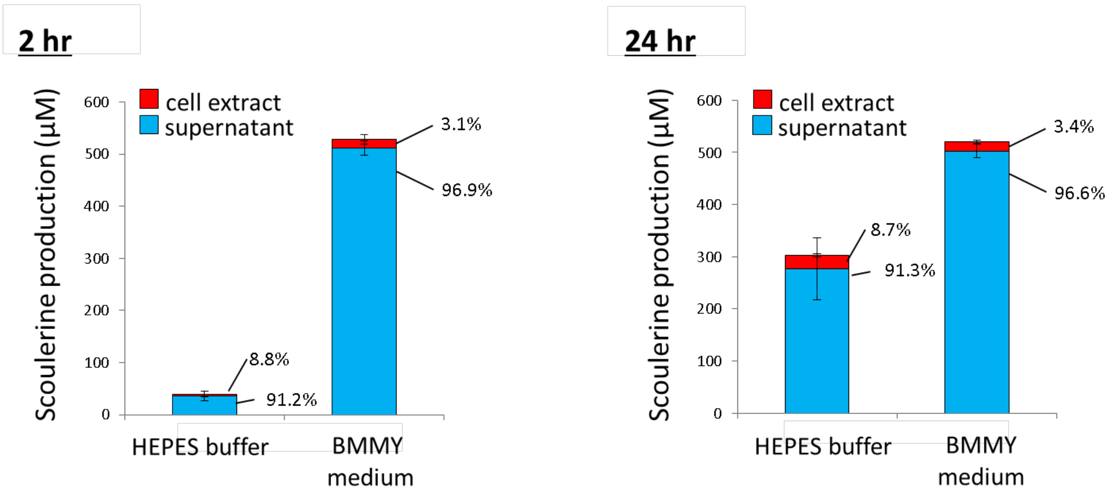

Supplementary Figure S3. Comparison of the effect of the reaction conditions on bioconversion activity; HEPES buffer vs BMMY medium.
The amount of scoulerine produced from 1 mM (*R, S*)-reticuline after 2 or 24 hr of incubation with *Pichia* cells expressing BBE in HEPES buffer or BMMY medium. Error bars indicate the standard deviation calculated from three independent experiments.

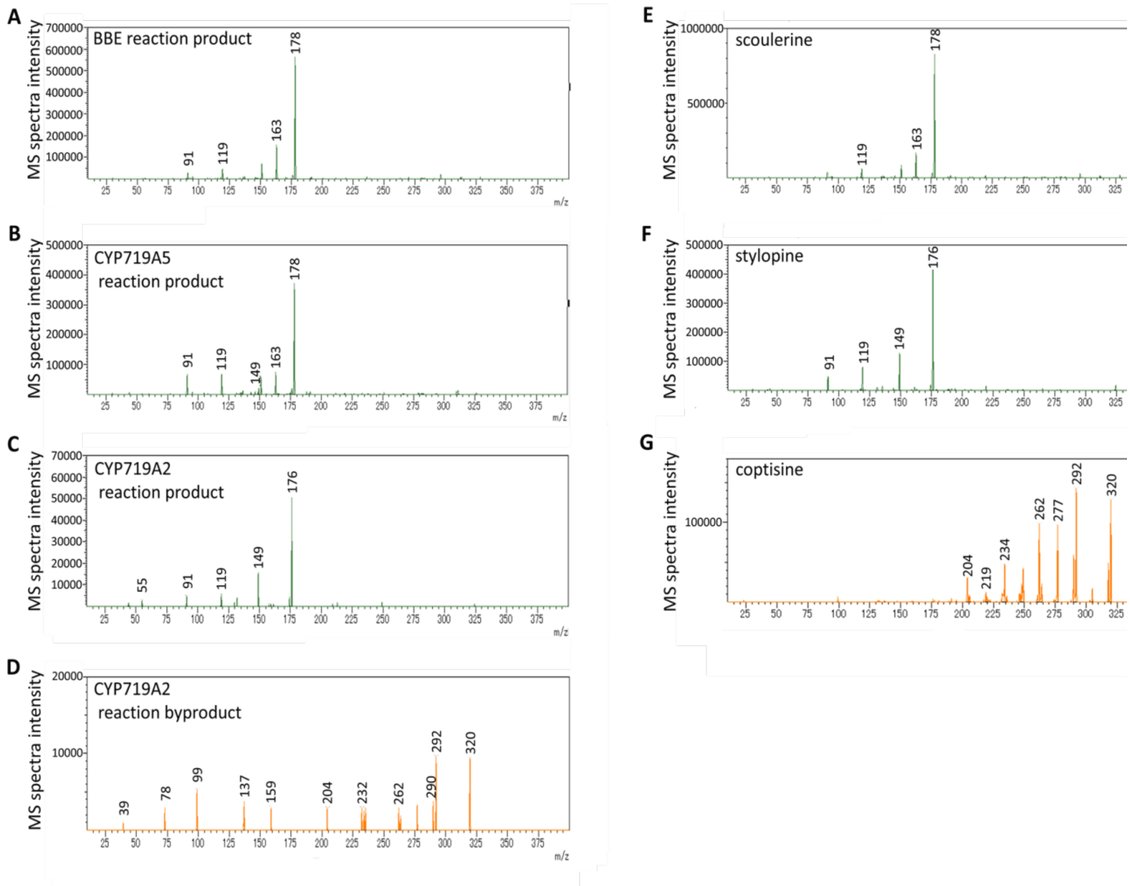

Supplementary Figure S4. MS fragments spectra of *in vivo* reaction products of BBE, CYP719A5, and CYP719A2 and authentic samples.
(A), BBE reaction product (precursor ion: m/z 328); (B), CYP719A5 reaction product (precursor ion: m/z 326); (C), CYP719A2 reaction product (precursor ion: m/z 324); (D), CYP719A2 reaction by-product (precursor ion: m/z 320); (E), authentic scoulerine (precursor ion: m/z 328, $[M+H]^+$); (F), authentic stylopine (precursor ion: m/z 324, $[M+H]^+$); (G), authentic coptisine (precursor ion: m/z 320, $[M]^+$).

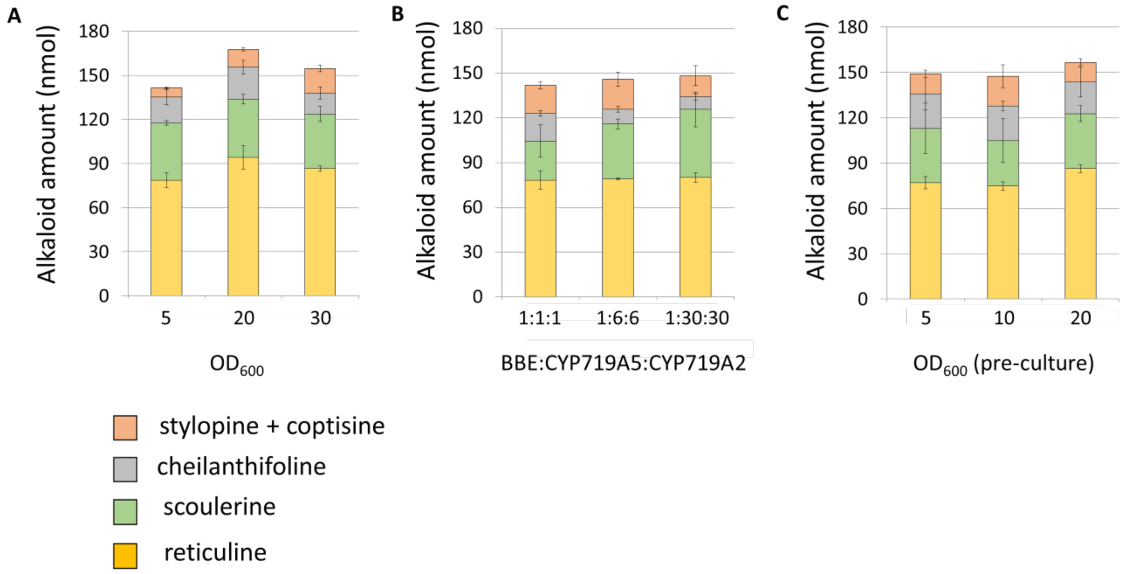

Supplementary Figure S5. Effects of different cell densities (A), ratios of transformants (B), or preculture conditions (C) on the bioconversion of reticuline in co-culture system

Bioconversion activities were determined as a whole reaction in a co-culture system after 24 hr of incubation; (A) Cell densities were modified from OD_{600} of 5 to 30 in the bioconversion reaction, after cells were pre-cultured until OD_{600} = 30. Three transformants (BBE:CYP719A5:CYP719A2) were mixed at 1:1:1. (B) Three transformants were mixed with different ratios (BBE:CYP719A5:CYP719A2 = 1:1:1, 1:6:6 and 1:30:30) at total OD_{600} = 30, after cells were grown until OD_{600} = 30. (C) After transformants were pre-cultured until OD_{600} = 5, 10 and 20, bioconversion activity of cells were measured at the cell density of OD_{600} = 30 with the ratio of 1:1:1 of the three transformants. The alkaloid amounts were determined by HPLC analysis at 280 nm. Error bars indicate the standard deviation calculated from three independent experiments.
